# Assessing Mental Health: Depressive Disorders among Healthcare Workers During Pre-Employment Evaluations

**DOI:** 10.1192/j.eurpsy.2026.11429

**Published:** 2026-07-31

**Authors:** N. Gannoun, N. Belhadj, R. Nakhli, F. Chelly, M. Makhloufi, M. Bouhoula, I. Kacem, A. Aloui, J. Ben Khelifa, C. Sridi, I. Fki, M. Maoua

**Affiliations:** 1 Occupational Medicine Department, Sahloul University Hospital; 2 Faculty of Medicine of Sousse, University of Sousse; 3 sousse, Research laboratory (LR19SP03); 4 Occupational Medicine Department, Farhat Hached University Hospital of Sousse; 5 Clinical Psychology Unit, Occupational Medicine Department, Sahloul University Hospital, sousse, Tunisia

## Abstract

**Introduction:**

Depressive disorders among healthcare professionals represent a significant occupational health issue. Pre-employment psychiatric assessments provide an opportunity to identify psychological vulnerabilities and ensure both professional efficiency and workplace safety.

**Objectives:**

This study aimed to describe depressive disorders and related psychosocial factors among healthcare workers during pre-employment evaluations.

**Methods:**

We conducted a descriptive cross-sectional study based on the medical records of healthcare workers undergoing mandatory pre-employment medical evaluations assessed in our Department of Occupational Medicine between 2024 and 2025. Each pre-employment evaluation included a clinical and paraclinical examination, along with a structured psychological interview assessing psychiatric history, current psychological state, and potential impact on professional performance. Medical history and previous treatment backgrounds were also systematically considered.

**Results:**

A total of six female healthcare workers were included, with a mean age of 35.3 years (range: 30–41). The professional positions included nurses, radiology technicians, and surgical instrumentation technicians. Among them, four patients reported a history of depressive episodes, including one case with previous hospitalization and suicide attempts. Two participants had received prior pharmacological treatment (bromazepam and lamotrigine). At the time of evaluation, three workers expressed demotivation or emotional fragility, whereas two presented no current symptoms despite a past psychiatric history. Technical and relational competencies were preserved in all cases. Five candidates were declared fit for work, with two requiring regular psychological follow-up due to stress vulnerability and risk of relapse. In one case, given the history of hospitalization and suicide attempts, a specialized psychiatric consultation was requested to provide a definitive opinion regarding professional fitness, and the decision was still pending at the time of reporting.

**Conclusions:**

**Discussion/Conclusion:** This study highlights the presence of depressive histories and psychological vulnerabilities among healthcare workers at the time of recruitment. Although all were declared fit for work, systematic psychiatric assessment during pre-employment evaluations appears essential. Early detection of at-risk individuals may guide preventive strategies and the implementation of tailored psychological support programs, thereby reducing relapse risk and improving resilience in demanding healthcare environments.

**Disclosure of Interest:**

None Declared

